# Peanut allergy: Effect of environmental peanut exposure in children with filaggrin loss-of-function mutations

**DOI:** 10.1016/j.jaci.2014.08.011

**Published:** 2014-10

**Authors:** Helen A. Brough, Angela Simpson, Kerry Makinson, Jenny Hankinson, Sara Brown, Abdel Douiri, Danielle C.M. Belgrave, Martin Penagos, Alick C. Stephens, W.H. Irwin McLean, Victor Turcanu, Nicolaos Nicolaou, Adnan Custovic, Gideon Lack

**Affiliations:** a Department of Pediatric Allergy, Division of Asthma, Allergy and Lung Biology, King's College London and Guy's and St. Thomas' NHS Foundation Trust, London, United Kingdom; b Centre for Respiratory Medicine and Allergy, Institute of Inflammation and Repair, Manchester Academic Health Sciences Centre, University of Manchester and University Hospital of South Manchester NHS Foundation Trust, Manchester, United Kingdom; c Centre for Health Informatics, Institute of Population Health, University of Manchester, Manchester, United Kingdom; d Centre for Dermatology and Genetic Medicine, College of Life Sciences and College of Medicine, Dentistry and Nursing, University of Dundee, Dundee, United Kingdom; e Department of Public Health Science, School of Medicine, King's College London, London, United Kingdom

**Keywords:** *FLG* loss-of-function mutations, filaggrin, skin barrier, peanut sensitization, peanut allergy, environmental peanut exposure, dust, threshold, AD, Atopic dermatitis, CRD, Component-resolved diagnostics, *FLG*, Filaggrin, GEE, Penalized generalized estimating equations methodology, ISU, ISAC standardized unit, LLQ, Lower limit of quantitation, MAAS, Manchester Asthma and Allergy Study, OFC, Oral food challenge, OR, Odds ratio, sIgE, Allergen-specific IgE, SPT, Skin prick test

## Abstract

**Background:**

Filaggrin *(FLG)* loss-of-function mutations lead to an impaired skin barrier associated with peanut allergy. Household peanut consumption is associated with peanut allergy, and peanut allergen in household dust correlates with household peanut consumption.

**Objective:**

We sought to determine whether environmental peanut exposure increases the odds of peanut allergy and whether *FLG* mutations modulate these odds.

**Methods:**

Exposure to peanut antigen in dust within the first year of life was measured in a population-based birth cohort. Peanut sensitization and peanut allergy (defined by using oral food challenges or component-resolved diagnostics [CRD]) were assessed at 8 and 11 years. Genotyping was performed for 6 *FLG* mutations.

**Results:**

After adjustment for infantile atopic dermatitis and preceding egg skin prick test (SPT) sensitization, we found a strong and significant interaction between natural log *(ln [loge])* peanut dust levels and *FLG* mutations on peanut sensitization and peanut allergy. Among children with *FLG* mutations, for each *ln* unit increase in the house dust peanut protein level, there was a more than 6-fold increased odds of peanut SPT sensitization, CRD sensitization, or both in children at ages 8 years, 11 years, or both and a greater than 3-fold increased odds of peanut allergy compared with odds seen in children with wild-type *FLG*. There was no significant effect of exposure in children without *FLG* mutations. In children carrying an *FLG* mutation, the threshold level for peanut SPT sensitization was 0.92 μg of peanut protein per gram (95% CI, 0.70-1.22 μg/g), that for CRD sensitization was 1.03 μg/g (95% CI, 0.90-1.82 μg/g), and that for peanut allergy was 1.17 μg/g (95% CI, 0.01-163.83 μg/g).

**Conclusion:**

Early-life environmental peanut exposure is associated with an increased risk of peanut sensitization and allergy in children who carry an *FLG* mutation. These data support the hypothesis that peanut allergy develops through transcutaneous sensitization in children with an impaired skin barrier.

There is a clear association between early-onset atopic dermatitis (AD) and food allergy.^1^, ^2^ Children with AD have an impaired skin barrier, which might allow antigen to penetrate the skin and sensitize the subject.^3^, ^4^ In children with a history of AD, 90% of those who went on to have peanut allergy had been exposed topically to creams containing *Arachis* species (peanut) oil in the first 6 months of life.^2^ In mice epicutaneous exposure to food allergens after skin stripping induces a potent allergic T_H_2-type response associated with high IL-4, IL-5, and allergen-specific IgE (sIgE) levels and systemic anaphylaxis after oral challenge.^5^, ^6^

Filaggrin is responsible for the strength and integrity of the stratum corneum^7^ and regulates the permeability of the skin to water and antigens.^8^ Loss-of-function mutations in the gene encoding filaggrin *(FLG)* are present in up to 50% of patients with moderate-to-severe AD^9^, ^10^ and have been shown to increase the risk of inhalant allergic sensitization, allergic rhinitis, asthma,^11^, ^12^ and peanut allergy.^13^ In the flaky tail mouse, which has a 1-bp deletion mutation (5303delA) within the murine *flg* gene (analogous to common human *FLG* loss-of-function mutations), topical allergen application leads to cellular infiltration and allergen-specific antibody response, even without skin stripping.^14^ This suggests that filaggrin deficiency, even in the absence of dermatitis, might be sufficient for transcutaneous sensitization.

High consumption of peanut by household members during the child's first year of life is associated with an increased risk of peanut allergy, possibly because of environmental peanut exposure in the child's home^15^; however, in this study questionnaire-based assessment of household peanut consumption was not validated against an objective measure of peanut in the environment and was potentially subject to retrospective bias. We recently showed that peanut protein in household dust is positively correlated with household peanut consumption.^16^ In addition, we showed that peanut protein in dust activates basophils from children with peanut allergy in a dose-dependent manner and is thus biologically active.^16^

We hypothesized that peanut sensitization can occur through presentation of environmental peanut antigen through an impaired skin barrier to underlying antigen-presenting cells. To address this hypothesis, we investigated whether early-life environmental peanut exposure measured directly by quantifying peanut antigen in household dust was a risk factor for the development of peanut allergy and whether this relationship was modified by *FLG* genotype. Specifically, we predicted that an increase in the peanut protein concentration in household dust during infancy would be associated with an increase in school-age peanut sensitization and allergy and that this effect would be augmented in children with 1 or more *FLG* loss-of-function mutations.

## Methods

### Study population

The Manchester Asthma and Allergy Study (MAAS) is an unselected birth cohort described in detail elsewhere (registration: ICRCTN72673620).^17^ In brief, 1184 subjects were recruited prenatally from 1995 to 1997 and followed up at ages 1, 3, 5, 8, and 11 years. The study was approved by the local ethics committee; parents provided written informed consent.

### Data sources

Validated questionnaires were interviewer administered to collect information on parentally reported symptoms and physicians' diagnoses. Parental report of a history of AD during infancy was assessed by using a modified International Study of Asthma and Allergies in Childhood questionnaire to apply the UK Working Party's diagnostic criteria for AD.^18^ Peanut sensitization was assessed at ages 8 and 11 years by using skin prick tests (SPTs) to whole peanut extract (Hollister-Stier, Spokane, Wash)^19^ and by measuring sIgE to whole peanut extract and peanut components Ara h 1, 2, and 3 with ImmunoCAP (age 8 years) or the ISAC Multiplex Immuno Solid-phase Allergen Chip (age 11 years; Thermo Fisher Scientific, Uppsala, Sweden).^20^ Maternal peanut consumption during pregnancy and breast-feeding were collected retrospectively (aged 8 years) in a subset of patients assessed for peanut allergy by means of diagnostic oral food challenge (OFC).

### Definition of outcomes

#### Peanut SPT sensitization

Peanut SPT sensitization was defined as a mean wheal diameter of 3 mm or greater than that elicited by the negative control.

#### Peanut component-resolved diagnostics sensitization

Peanut component-resolved diagnostics (CRD) sensitization was defined as sIgE to the peanut components Ara h 1, 2, or 3 of 0.35 kU_A_/L or (8 years) or 0.35 ISAC standardized units (ISU) or greater (11 years).^20^ Patients with Ara h 1, 2, or 3 levels of less than 0.35 kU_A_/L (8 years) or 0.35 ISU (11 years) were deemed non-CRD sensitized. If no CRD analysis was available, then patients with peanut sIgE levels of less than 0.2 kU_A_/L ImmunoCAP were considered not CRD sensitized.

#### Peanut allergy

All children with evidence of peanut sensitization at age 8 years (peanut SPT response ≥3 mm or sIgE level ≥0.2 kU_A_/L) were offered an OFC to peanut to determine allergy versus tolerance.^19^ Open OFCs were applied among children who had a history of tolerating peanut on consumption; all other children underwent a double-blind, placebo-controlled OFC.^19^ OFC results were considered positive after development of 2 or more objective signs indicating an allergic reaction.^19^ Children with a convincing history of an immediate hypersensitivity reaction on exposure to peanut combined with a peanut sIgE level of 15 kU_A_/L or greater,^21^ an SPT response of 8 mm or greater,^22^ or both (age 8 years) were considered to have peanut allergy and did not undergo an OFC. Two children with a convincing history of an immediate hypersensitivity reaction on exposure to peanut and an SPT response of 3 mm or greater who refused consent for OFCs were considered to have peanut allergy based on an Ara h 2 level of 0.35 ISU or greater^19^ at subsequent follow-up at age 11 years.

### Quantitation of environmental peanut exposure in household dust

Dust samples were collected predominantly at 36 weeks' gestation from the lounge-sofa, as previously described.^23^ If no antenatal dust sample was available from the lounge-sofa, then dust samples from 6 or 12 months were analyzed for peanut protein (where available). Dust samples were extracted in borate-buffered saline (0.1% Tween 20, pH 8.0) and stored at −20°C until analysis. Peanut protein in dust extracts was determined by using the Veratox polyclonal ELISA against whole peanut protein (Neogen, Lansing, Mich), which has been validated for sensitivity, specificity, and reliability in measuring peanut protein contamination of food,^24^, ^25^ dust, and wipe samples.^26^ The Veratox ELISA lower limit of quantitation (LLQ) for peanut protein in dust was 100 ng/mL (0.5 μg/g based on a dust sample weighing between 50-100 mg); this variable was analyzed by using a fixed calculation for values of less than this level (LLQ/2; results are shown in Table E1 in this article's Online Repository at www.jacionline.org)^27^ and by using all data of less than this value (results in the main body of the article) because the variable with LLQ/2 created 230 (37%) censored data points.^28^ Analyses for both forms of the peanut dust variable were compared to determine whether the 2 different ways of dealing with data of less than the LLQ made a material difference to the results obtained. Participant information was blinded from the researcher performing the ELISA-based dust analyses.

### Genotyping

*FLG* genotyping was performed with probes and primers, as previously described.^9^ Genotyping for R501X, S3247X, and R2447X loss-of-function mutations was performed with a TaqMan-based allelic discrimination assay (Applied Biosystems, Cheshire, United Kingdom). Mutation 2282del4 was genotyped by sizing of a fluorescently labeled PCR fragment on a 3100 or 3730 DNA sequencer. *FLG* mutations 3673delC and 3702delG were assessed by means of GeneScan analysis of fluorescently labeled PCR products. These 6 *FLG* mutations have been consistently associated with AD in white populations^10^; however, because some of these *FLG* mutations are not found in nonwhite subjects,^29^ all nonwhite participants were excluded from analyses that included *FLG* genotype. Data were analyzed as combined carriage of an *FLG* null allele; that is, if a child carried 1 or more of the 6 genetic variations, he or she was considered an *FLG* null allele carrier. Complete *FLG* genotype results (ie, results for all 6 *FLG* loss-of-function mutations screened) were available for 805 (76.0%) of 1059 white participants, 117 samples failed genotype analysis for 1 or more mutations, and no sample was available in 137 participants. In cases with incomplete *FLG* data, the presence of 1 *FLG* mutation defined that case as a carrier; participants with incomplete genotyping data in whom all successfully tested alleles were wild-type alleles were excluded from further analysis because their *FLG* genotype status remained ambiguous.

### Statistical analysis

Data were analyzed with STATA 12.1 software (StataCorp, College Station, Tex). Demographics and clinical characteristics were compared between participants and nonparticipants. Count data were compared by using the Pearson χ^2^ test. Continuous data were compared with the Student *t* test for normally distributed data and the Mann-Whitney *U* test for nonnormally distributed data. All variables except maternal age and peanut protein in dust were compared by using the Pearson χ^2^ test. Maternal age was normally distributed and thus was compared with the Student *t* test. Peanut protein in dust (without natural log *[ln]* transformation) was not normally distributed and thus was compared with the Mann-Whitney *U* test. Peanut protein in dust (in micrograms per gram) underwent *ln* transformation for subsequent analyses. Factors associated with peanut allergy at the ages of 8 years, 11 years, or both were assessed by using a penalized logistic regression methodology to account for unbalanced data (20/577 had peanut allergy).^30^ Factors associated with peanut sensitization (SPT and CRD results) were assessed by using penalized generalized estimating equations methodology (GEE) through a quasi–least squares approach, with an exchangeable working correlation matrix to account for repeated measures within subjects at 8 and 11 years.^31^ Goodness of fit of the GEE statistical model was assessed by using the quasilikelihood under independence model criterion. The goodness of fit of the penalized logistic regression methodology statistical model was assessed by using the Akaike information criterion. We tested whether the effect of environmental peanut exposure on peanut sensitization and allergy was modified by *FLG* genotype by including an interaction term.

The additive effect of *FLG* loss-of-function mutation was calculated by using the exponential of the coefficient (β) of the interaction (*FLG* genotype by peanut dust exposure) minus the baseline coefficient (β) of peanut dust exposure. The predictive probability of peanut sensitization and allergy was calculated from the multivariate regression model. Threshold levels of peanut protein in dust for peanut sensitization and allergy were calculated by using the intersection between wild-type *FLG* versus *FLG* mutation in the multivariate regression model.^30^, ^32^ To evaluate the reliability of the thresholds obtained and the uncertainty around them, we conducted bootstrap cross-validation with 1000 replications.

## Results

### Participants and descriptive data

**Fig 1:**
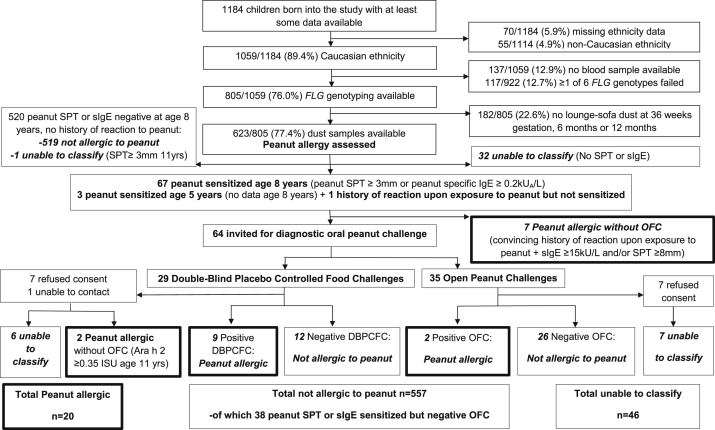
CONSORT diagram outlining participant flow. Peanut allergy outcomes are highlighted in *boxes outlined in boldface*. *DBPCFC*, Double-blind, placebo-controlled food challenge.

Details of the participant flow are presented in Fig 1. From 1184 participants, we analyzed data from 623 white children with available *FLG* genotyping and early-life environmental peanut exposure. Of these children, at age 8 years, 32 had no peanut SPT or peanut sIgE data, 70 were peanut sensitized (of these, 3 children were sensitized at age 5 years and had no peanut SPT or sIgE data at age 8 years), 1 was not peanut sensitized but reported a reaction on peanut exposure, and 520 were not peanut sensitized and reported no reactions to peanut (of these, 1 was subsequently peanut sensitized at age 11 years and thus impossible to classify). Seven children with a convincing history of an allergic reaction on peanut exposure and a peanut sIgE level of 15 kU_A_/L or greater, an SPT response of 8 mm or greater, or both were classified as having peanut allergy; the remaining 64 sensitized children were invited for an OFC (29 double-blind, placebo-controlled food challenges and 35 open challenges). We were unable to contact 1 subject, and 14 refused consent (of these, 2 were classified as having peanut allergy at age 11 years on the basis of a convincing history of an immediate hypersensitivity reaction on exposure to peanut and an Ara h 2 level ≥0.35 ISU). Thus 20 children were defined as having peanut allergy, 557 were defined as nonallergic, and 46 could not be classified (because of missing SPT and sIgE data or because they declined consent for an OFC).

The demographics of the whole group, both included and excluded children, are shown in Table I. Comparison of the included and excluded groups revealed no differences in peanut sensitization or allergy; we observed small (but statistically significant) differences in parental atopy, *FLG* status, history and severity of AD, sex, breast-feeding, and sibship position. *FLG* loss-of-function mutations were carried by 57 (9.1%) of 623 children (all children; Table I) and 4 (20%) of 20 children with peanut allergy (Table II). A history of infantile AD was present in 207 (33.7%) of 614 (all children) children and 16 (80%) of 20 children with peanut allergy. Of the 16 children with peanut allergy with wild-type *FLG*, 13 (81%) had a history of infantile AD. The median peanut protein concentration in dust was 0.73 μg/g (interquartile range, 0.40-1.33 μg/g); the peanut allergen level was less than the LLQ in 230 (36.9%) of 623 homes.

**Table I:** Demographics and clinical characteristics of the included group (n = 623) versus the excluded group (n = 561) and whole group (n = 1184)

	Included group∗ (n = 623)		Excluded group† (n = 561)		Whole group (n = 1184)		*P* value, included (n = 623) vs excluded (n = 561)
	Total no.	No. (%)	Total no.	No. (%)	Total no.	No. (%)	
Peanut SPT sensitization at 8 y	559	30 (5.4)	360	18 (5.0)	920	48 (5.2)	.69
Peanut SPT sensitization at 11 y	450	19 (4.2)	256	13 (5.1)	706	32 (4.5)	.41
Peanut SPT sensitization at age 8 and/or 11 y§	434	35 (8.1)	237	24 (10.1)	710	59 (8.3)	.15
Peanut CRD sensitization at age 8 y	371	13 (3.5)	211	7 (3.3)	584	20 (3.4)	.84
Peanut CRD sensitization at age 11 y	297	12 (4.0)	154	8 (5.2)	451	20 (4.4)	.37
Peanut CRD sensitization at age 8 and/or 11 y§	241	19 (7.9)	116	9 (7.8)	357	28 (7.8)	.94
Peanut allergy at age 8 and/or 11 y	577	20 (3.5)	382	10 (2.6)	959	30 (3.1)	.19
History of AD during infancy	614	207 (33.7)	477	190 (39.8)	1091	397 (36.4)	<.01
No AD on clinical assessment at age 1 y	338	272 (80.5)	173	142 (82.1)	511	414 (81.0)	.46
Mild AD on assessment at age 1 y	338	46 (13.6)	173	25 (14.5)	511	71 (13.9)	.66
Moderate/severe AD at age 1 y	338	20 (5.9)	173	6 (3.5)	511	26 (5.1)	.01
Combined *FLG* loss-of-function mutation	623	57 (9.1)	234	29 (12.4)	857	86 (10.0)	.02
Parental report of “hay fever ever” in the child	569	135 (23.7)	400	105 (26.3)	969	240 (24.8)	.18
Egg SPT sensitization at age 3 y	545	21 (3.9)	398	15 (3.8)	943	36 (3.8)	.92
Male sex	623	311 (49.9)	561	331 (59.0)	1184	642 (54.2)	<.001
Full older siblings (same mother and father)	623	316 (50.7)	532	297 (55.8)	1155	614 (53.2)	.02
Parental atopy (low vs medium/high risk)	621	501 (80.7)	514	443 (86.2)	1135	944 (83.2)	.001
Breast-feeding (yes vs no)	618	443 (71.7)	497	337 (67.8)	1115	780 (70.0)	.03
Peanut consumption during pregnancy (yes vs no)	70	56 (80.0)	41	35 (85.4)	111	91 (82.0)	.28
Peanut consumption during breast-feeding (yes vs no)	59	45 (76.3)	29	24 (82.8)	88	69 (78.4)	.26
House dust mite reduction measures‡	160	88 (55.0)	93	45 (48.4)	253	133 (52.6)	1.00
Maternal age at baseline (y), mean (SD)	615	30.67 (4.74)	499	30.02 (4.81)	1114	30.38 (4.78)	.51
Peanut protein in dust (μg/g) using values below LLQ, median (IQR)	623	0.73 (0.40-1.33)	128	0.78 (0.36-1.40)	751	0.73 (0.38-1.35)	.96
Peanut protein in dust (μg/g) using LLQ/2, median (IQR)	623	0.73 (0.25-1.33)	128	0.78 (0.25-1.40)	751	0.73 (0.25-1.35)	.90

*IQR*, Interquartile range.

∗ Included group comprised of white children enrolled in MAAS with available sofa dust within the first year of life and successful *FLG* genotyping.

† Children were excluded for the following reasons: (1) nonwhite ethnicity, (2) lack of available blood sample for *FLG* genotyping or failed genotyping, or (3) no dust extract available for the assessment of environmental peanut allergen exposure.

‡ “High-risk” infants (both parents with positive SPT responses) with no pets in the home in MAAS were randomized to house dust mite reduction measures versus control subjects.

§ Children who were not peanut sensitized at age 8 or 11 years and missing data at the other time point were classed as having missing sensitization data.

**Table II:** *FLG* genotype frequencies in 20 children with peanut allergy and 577 children without peanut allergy at ages 8 years, 11 years, or both

	R501X	2282del4	S3247X	R2447X	3673delC	3702delG	Combined *FLG* loss-of-function genotype
No. (%) of peanut allergic children with *FLG* genotype (n = 20)							
Wild-type *FLG*	18 (90.0)	17 (85.0)	0 (0.0)	0 (0.0)	0 (0.0)	0 (0.0)	16 (80.0)
*FLG* loss-of-function mutation	2 (10.0)	3 (15.0)	0 (0.0)	0 (0.0)	0 (0.0)	0 (0.0)	4 (20.0)∗†
Failure of analysis‡	0 (0.0)	0 (0.0)	0 (0.0)	0 (0.0)	0 (0.0)	0 (0.0)	

No. (%) of children without peanut allergy with *FLG* genotype (n = 557)							
Wild-type *FLG*	533 (95.7)	535 (96.05)	552 (99.1)	551 (98.9)	603 (100.0)	603 (100.0)	507 (91.0)
*FLG* loss-of-function mutation	23 (4.1)	20 (3.6)	4 (0.7)	5 (0.9)	0 (0.0)	0 (0.0)	50 (9.0)∗§
Failure of analysis‡	1 (0.2)	2 (0.35)	1 (0.2)	1 (0.2)	0 (0.0)	0 (0.0)	

∗ There were no mutant allele homozygotes for any allele tested.

† This includes 1 compound heterozygote (R501X/2282del4).

‡ Although individual *FLG* genotypes failed, if a child had incomplete data but had a mutant *FLG* allele, they were included as a case in the combined loss-of-function genotype. If they had incomplete data but all alleles successfully tested were wild-type alleles, they were excluded because this could indicate a false-negative result.

§ This includes 2 compound heterozygotes (R501X/2282del4).

### *FLG* genotype modifies the effect of early-life environmental peanut on the risk of peanut sensitization and allergy

**Fig 2:**
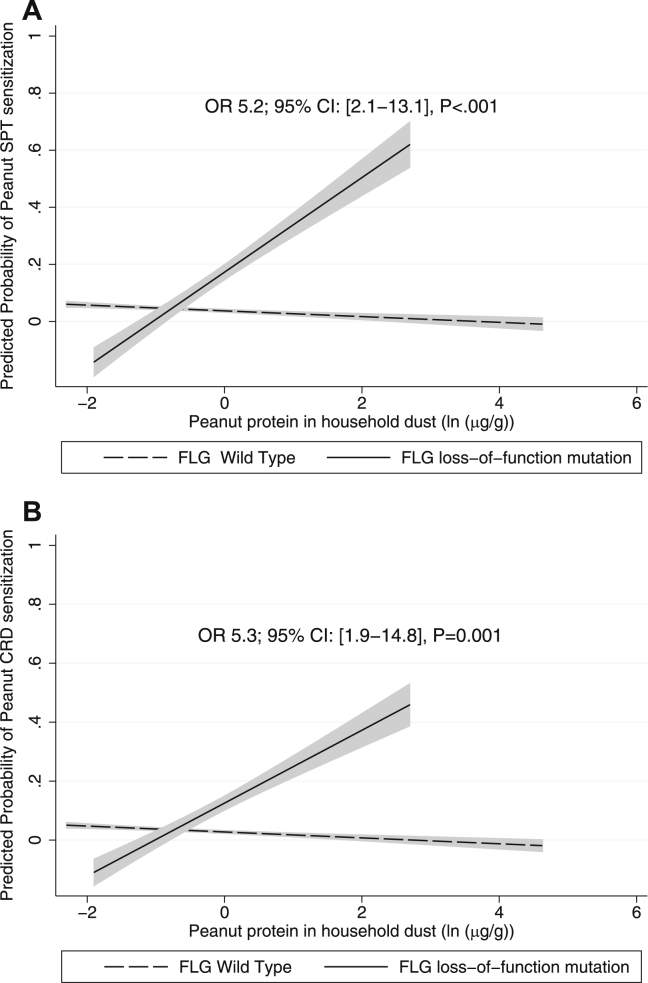
Mean predictive probability of peanut sensitization over 8 and 11 years on GEE analysis with increasing environmental peanut exposure (defined by *ln* transformed peanut protein in micrograms per gram of dust) for children with 1 or more *FLG* loss-of-function mutations versus those with wild-type *FLG*. The model was adjusted for a history of infantile AD and egg SPT sensitization at age 3 years. Interaction ORs and 95% CIs displayed between peanut protein in dust and *FLG* loss-of-function mutations on peanut sensitization are shown. Predictive probability is only shown within the observable environmental peanut exposure data obtained. **A,** Peanut SPT sensitization. **B,** Peanut CRD sensitization.

**Fig 3:**
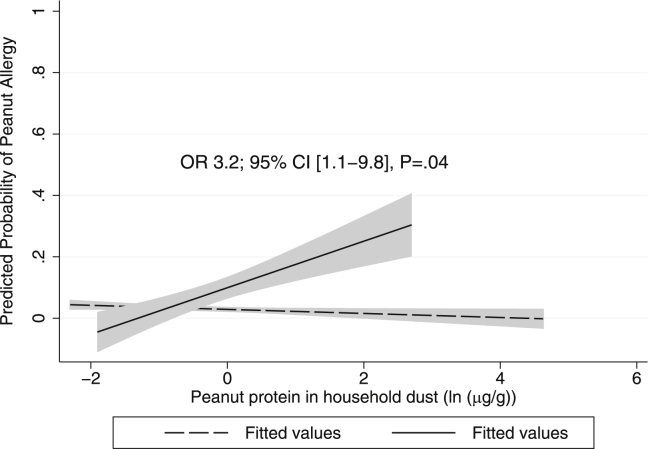
Mean adjusted predictive probability of peanut allergy at 8 years, 11 years, or both on multivariate penalized logistic regression analysis with increasing environmental peanut exposure (defined by *ln* transformed peanut protein in micrograms per gram of dust) in children with 1 or more *FLG* loss-of-function mutations versus those with wild-type *FLG*. Interaction ORs and 95% CIs are displayed between peanut protein in dust and *FLG* loss-of-function mutations on peanut allergy. Predictive probability is only shown within the observable environmental peanut exposure data obtained.

Factors associated with both peanut sensitization and peanut allergy were history and severity of infantile AD, *FLG* loss-of-function mutation (trend for allergy), egg SPT sensitization at age 3 years, and parental report of “hay fever ever” in the child on univariate analysis (Table III). Peanut protein levels in dust were not associated with peanut sensitization or allergy overall; however, there was a strong and significant interaction on univariate analysis between *FLG* genotype and early-life environmental peanut exposure on peanut SPT sensitization (odds ratio [OR], 5.3; 95% CI, 1.8-15.3; *P* < .01) and peanut CRD sensitization (OR, 4.5; 95% CI, 1.5-13.5; *P* < .01) and a trend toward peanut allergy (OR, 2.7; 95% CI, 0.9-8.0; *P* = .07) (Table IV). Given the low number of children with peanut allergy outcomes, we were conservative in the selection of covariates in the multivariate model and used 2 covariates (egg SPT sensitization at age 3 years and a history of infantile AD) that were both highly associated with peanut SPT/CRD sensitization and allergy. In the multivariate analysis, with the inclusion of an interaction variable for *FLG* genotype*(*ln* peanut exposure), we found a strong and significant change in *FLG* genotype divergence with early-life environmental peanut exposure on both peanut sensitization and allergy (Table IV). These interactions were consistent for peanut SPT sensitization (OR, 5.2; 95% CI, 2.1-13.1; *P* < .001; Fig 2, *A*), peanut CRD sensitization (OR, 5.3; 95% CI, 1.9-14.8; *P* = .001; Fig 2, *B*), and clinically confirmed peanut allergy (OR, 3.2; 95% CI, 1.1-9.8; *P* = .04; Fig 3). Analysis of the peanut dust variable with LLQ/2 did not show a material difference in results (see Table E1). The additive effect of each *ln* unit increase in house dust peanut in children with 1 or more *FLG* loss-of-function mutations was 6.1-fold for peanut SPT sensitization, 6.5-fold for peanut CRD sensitization, and 3.3-fold for peanut allergy in the multivariate model. In children with a wild-type *FLG* genotype, there was no association between early-life environmental peanut exposure and subsequent peanut sensitization or allergy.

**Table III:** Clinical and demographic factors associated with peanut SPT and CRD sensitization and peanut allergy on univariate GEE and penalized logistic regression methodology analysis

	Peanut SPT sensitization adjusted for age at assessment (8 + 11 y; GEE; n = 584)			Peanut CRD sensitization adjusted for age at assessment (8 + 11 y; GEE; n = 437)			Peanut allergy at age 8 y, 11 y, or both (LR; n = 577)		
	OR	95% CI	*P* value	OR	95% CI	*P* value	OR	95% CI	*P* value
History of AD during infancy	10.5	4.2-26.1	**<.001**	11.9	3.3-43.1	**<.001**	8.9	2.9-26.9	**<.001**
AD severity, no AD at 1 y	Reference category			Reference category			Reference category		
Mild AD on assessment at 1 y	2.2	0.6-8.4	.25	3.4	0.7-16.5	.13	5.0	1.1-23.2	**.04**
Moderate-to-severe AD at 1 y	20.8	4.1-62.4	**<.001**	16.6	3.2-86.6	**.001**	28.0	6.6-118.8	**<.001**
Combined *FLG* loss-of-function mutations	3.5	1.5-8.3	**<.01**	4.0	1.4-11.4	**<.01**	2.5	0.8-7.9	.11
Parental report of “hay fever ever” in the child	3.4	1.6-7.3	**.001**	3.4	1.3-9.2	**.02**	4.2	1.6-11.1	**<.01**
Egg SPT sensitization at age 3 y	12.3	4.5-33.6	**<.001**	16.4	4.8-56.0	**<.001**	25.5	8.4-77.0	**<.001**
Male sex	2.2	1.0-4.6	**.04**	1.8	0.7-4.8	.22	1.6	0.6-3.9	.33
Full older siblings (same mother and father)	0.9	0.4-1.8	.72	0.5	0.2-1.4	.19	0.7	0.3-1.8	.46
Parental atopy, low vs medium/high risk	6.9	0.9-51.4	.06	1.9	0.4-8.3	.42	4.7	0.6-35.5	.13
Breast-feeding (yes vs no)	1.0	0.5-2.2	.99	2.7	0.6-11.9	.19	1.6	0.5-4.8	.43
Peanut consumption during pregnancy (yes vs no)	1.0	0.3-2.8	.93	0.8	0.2-2.8	.72	0.5	0.2-1.9	.32
Peanut consumption during breast-feeding (yes vs no)	0.8	0.3-2.3	.65	0.8	0.2-2.7	.70	0.6	0.2-2.0	.38
House dust mite reduction measures	1.0	0.3-3.2	.95	0.8	0.2-4.4	.81	0.7	0.1-2.6	.57
Maternal age at baseline (y)	1.0	1.0-1.1	.31	1.1	1.0-1.1	.06	1.0	0.9-1.1	.79
Peanut protein in dust (*ln* transformed μg/g)∗	1.3	0.9-1.7	.16	1.2	0.8-1.8	.33	1.2	0.8-1.8	.47
Age at assessment (8 or 11 y)	0.8	0.5-1.1	.10	1.0	0.7-1.5	1.00	NA	NA	NA

Values in boldface are significant.

*LR*, Penalized logistic regression methodology; *NA*, not applicable.

∗ Peanut protein in dust: values less than the LLQ were used in this analysis.

**Table IV:** GEE for peanut sensitization using quasilikelihood under independent model criterion goodness-of-fit analyses

	GEE peanut SPT sensitization adjusted for clustering at age 8 + 11 y (n = 584)					GEE peanut CRD sensitization adjusted for clustering at age 8 + 11 y (n = 437)					LR for peanut allergy at age 8 y, 11 y, or both (n = 577)				
	No.∗	OR	95% CI	*P* value	QIC§	No.†	OR	95% CI	*P* value	QIC§	No.‡	OR	95% CI	*P* value	AIC§
Combined *FLG* loss-of-function mutation	584	3.5	1.5-8.3	**<.01**	386.6	437	4.0	1.4-11.4	**<.01**	215.3	577	2.54	0.82-7.88	.11	175.6
Age at assessment (8 or 11 y)		0.8	0.5-1.1	.10			0.9	0.6-1.4	.69			NA			

Combined *FLG* loss-of-function mutation	584	3.6	1.5-8.2	**<.01**	386.7	437	4.0	1.4-11.0	**<.01**	216.5	577	2.5	0.8-7.9	.11	177.1
Peanut protein in dust (*ln* transformed μg/g)‖		1.3	0.9-1.7	.15			1.2	0.8-1.8	.27			1.2	0.8-1.8	.46	
Age at assessment (8 or 11 y)		0.7	0.5-1.1	.10			0.9	0.6-1.4	.66			NA			

Combined *FLG* loss-of-function mutation	584	2.4	0.7-8.6	.17	370.7	437	2.6	0.6-11.0	.20	207.9	577	2.2	0.6-8.2	.23	175.6
Peanut protein in dust (*ln* transformed μg/g)‖		0.9	0.6-1.3	.52			0.8	0.5-1.4	.38			0.9	0.6-1.6	.82	
Interaction FLG*peanut in dust		5.3	1.8-15.3	**<.01**			4.5	1.5-13.5	**<.01**			2.70	0.9-8.0	.07	
Age at assessment (8 or 11 y)		0.7	0.5-1.1	.10			0.9	0.6-1.4	.66			NA			

Combined *FLG* loss-of-function mutation	516	1.8	0.4-7.5	.41	303.9	396	1.3	0.2-7.6	.78	176.7	511	1.1	0.3-5.2	.87	132.5
Peanut protein in dust (*ln* transformed μg/g)‖		0.9	0.6-1.3	.50			0.8	0.5-1.5	.53			0.98	0.5-1.9	.98	
Interaction FLG*peanut in dust		6.8	2.6-17.5	**<.001**			6.6	2.3-18.9	**<.001**			3.9	1.3-11.8	**.02**	
Egg SPT sensitization at age 3 y		16.2	4.5-59.0	**<.001**			25.1	5.2-122.1	**<.001**			34.84	9.9-122.4	**<.001**	
Age at assessment (8 or 11 y)		0.7	0.5-1.1	.14			0.9	0.6-1.6	.82			NA			

Combined *FLG* loss-of-function mutation	516	1.1	0.3-5.2	.87	279.4	396	1.0	0.2-5.5	.95	167.6	511	0.8	0.2-3.9	.83	129.3
Peanut protein in dust (*ln* transformed μg/g)‖		0.9	0.6-1.3	.45			0.8	0.5-1.4	.46			1.0	0.5-1.8	.95	
Interaction FLG*peanut in dust		5.2	2.1-13.1	**<.001**			5.3	1.9-14.8	**.001**			3.2	1.1-9.8	**.04**	
Egg SPT sensitization at age 3 y		8.8	2.2-34.5	**<.01**			13.0	2.3-75.3	**<.01**			19.95	5.4-74.0	**<.001**	
History of AD during infancy		7.5	2.4-23.2	**<.001**			5.4	1.2-24.2	**.03**			4.04	1.2-14.1	**.03**	
Age at assessment (8 or 11 y)		0.7	0.4-1.1	.12			1.0	0.6-1.7	.90			NA			

Values in boldface are significant.

*AIC*, Akaike information criterion; *LR*, penalized logistic regression methodology; *NA*, not applicable; *QIC*, quasilikelihood under independent model criterion.

∗†‡ White children enrolled in MAAS with available sofa dust within the first year of life, successful *FLG* genotyping, and peanut SPT* or CRD† sensitization or peanut allergy‡ assessment.

§ Reductions in quasilikelihood under independent model criterion (GEE) and Akaike information criterion (LR) values denote improved goodness of fit of the statistical model.

‖ Peanut protein in dust: values less than the LLQ were used in this analysis.

### Threshold environmental peanut levels in dust for peanut sensitization and allergy

In children carrying 1 or more *FLG* loss-of-function mutations, the threshold environmental peanut allergen level for peanut SPT sensitization was −0.079 *ln* transformed units (0.92 μg of peanut protein/gram of dust; 95% CI, 0.70-1.22 μg/g), that for CRD sensitization was 0.032 *ln* transformed units (1.03 μg/g; 95% CI, 0.90-1.82 μg/g), and that for peanut allergy was 0.156 *ln* transformed units (1.17 μg/g; 95% CI, 0.01-163.83 μg/g).

## Discussion

This study demonstrates a gene-environment interaction on the development of peanut sensitization and clinically proven peanut allergy. In children carrying 1 or more *FLG* loss-of-function mutations, there was a dose-response relationship between early-life environmental exposure to peanut protein in household dust and subsequent peanut sensitization and allergy; each *ln* unit (2.7-fold) increase in house dust peanut exposure during infancy was associated with a more than 6-fold increase in the odds of school-age peanut sensitization and a 3.3-fold increase in the odds of school-age peanut allergy. Therefore we demonstrated a consistent interaction between *FLG* genotype and peanut dust exposure for peanut SPT sensitization, major allergen sensitization, and clinically proven peanut allergy. Previous studies have also shown a stronger effect of *FLG* loss-of-function mutations on peanut sensitization than peanut allergy.^33^ The interaction between *FLG* genotype and environmental peanut exposure was significant after adjusting for infantile AD and preceding egg sensitization; thus the modifying effect of *FLG* genotype was independent of AD or other atopy markers.

Among *FLG* mutation carriers, peanut protein levels in dust reached a maximum of 14.78 μg/g; thus an increase in peanut dust exposure from the LLQ (0.5 μg/g) to 14.78 μg/g equated to an almost 30-fold increase (3.4 *ln* scales), which is equivalent to a 58-fold (3.3^3.4^) increase in the odds of peanut allergy. These results suggest that the level of early-life environmental peanut exposure in children who carry *FLG* loss-of-function variants might critically influence the development of peanut sensitization and, importantly, clinical peanut allergy; however, future work is required to ensure the linearity of peanut protein data over the entire range of peanut protein in dust. In contrast, no association was seen between environmental peanut exposure and peanut sensitization or allergy in children without *FLG* mutations. In children carrying an *FLG* mutant allele, the mean threshold peanut protein level in dust for peanut sensitization and allergy was around twice the LLQ of the ELISA (0.50 μg/g). Thus on the basis of our findings in this white United Kingdom population, minimal quantities of peanut protein in the environment could lead to peanut sensitization and allergy in children who carry *FLG* loss-of-function mutations, but the risk markedly increases with increasing exposure.

Previous studies have shown gene-environment interactions between *FLG* loss-of-function mutations and other atopic diseases.^34^ Among children carrying an *FLG* mutation, those whose families owned a cat had an approximately 4-fold odds of having AD compared with those whose families did not own a cat; there was no effect of cat ownership among children without *FLG* mutations.^34^ Contact allergy to nickel is twice as common in adults with the *FLG* frameshift mutation 2282del4,^35^ and in murine models *flg* loss-of-function mutations lead to increased bidirectional paracellular penetration of water-soluble tracers and reduced inflammatory threshold to allergens.^36^ There is a significant association between *FLG* mutations and development of asthma and allergic sensitization but only in children with preceding AD.^37^ This has been used as an argument for the role of *FLG* loss-of-function mutations as a predisposing factor for allergic sensitization after epicutaneous exposure to allergens. Peanut protein in environmental dust and surfaces could penetrate disrupted skin because of impaired filaggrin production and could be taken up by Langerhans cells, leading to a T_H_2 response and IgE production by B cells.^38^, ^39^ Studies are investigating the role of thymic stromal lymphopoietin produced by keratinocytes in response to environmental antigens in patients with AD.^40^ Thymic stromal lymphopoietin in combination with enhanced allergen penetration through a damaged epidermis could lead to a T_H_2-type milieu; it would be interesting to review this in the context of filaggrin-deficient children with high levels of environmental peanut exposure.

There are certain limitations to this study. We were unable to include all MAAS participants because of the availability of early-life dust samples and *FLG* genotyping. Because the 6 *FLG* loss-of-function mutations assessed have been associated with AD in white populations,^12^ we excluded all nonwhite participants. Given that 95% of MAAS participants were white, this is unlikely to lead to bias. On comparing the groups of included versus excluded children, there were some small differences in their demographic characteristics, but importantly, there were no significant differences in peanut sensitization or allergy rates; therefore these are unlikely to have influenced the results. Peanut allergen levels in lounge-sofa dust might not be the best index of infant exposure; however, we have shown previously that there is high within-home correlation of peanut protein levels in dust, particularly between an infant's bed and play area.^26^ In our previously published work the infant play area was usually in the lounge, which was also the location of the sofa in the MAAS study. There were no available data on the amount of peanut the infant was consuming; however, given that the majority of dust collected was antenatal, these peanut dust levels would not have been due to the infant consuming peanut.

We acknowledge that there are small numbers of subjects with confirmed peanut allergy in whom *FLG* genotype and early-life peanut exposure data are available. This reflects the complexities of measuring all necessary predictors over the life course in children with robustly ascertained clinical outcomes that are themselves relatively uncommon (*FLG* loss-of-function mutations and clinical peanut allergy). We emphasize that the findings of an interaction between *FLG* loss-of-function genotype and environmental peanut exposure for sensitization (however measured) and peanut allergy are consistent, in keeping with previous gene-environment interactions for *FLG*, and biologically plausible.

It is important to consider how peanut allergen in dust might lead to sensitization to assess the clinical applicability of our findings; although this might lead to epicutaneous sensitization through direct skin contact, we cannot exclude the possibility of inhalation of dust particles containing peanut allergen. Although filaggrin is not expressed in the lung^41^ or inferior nasal turbinates,^42^ it is expressed in the cornified epithelium in the vestibular nasal lining.^11^ However, several studies suggest that peanut is poorly aerosolizeable^26^, ^43^ and report that allergic symptoms after inhalation of peanut have not been replicated on blinded challenges.^44^ It is also important to determine how peanut protein gets into household dust. Peanut protein is present on hand wipes and in saliva up to 3 hours after peanut consumption and thus might be amenable to transfer through this route.^26^ Fox et al^15^ found that household consumption of peanut butter was more highly associated with peanut allergy in infants than household consumption of covered forms of peanut-containing foods. They hypothesized that peanut butter was more likely to lead to sensitization through hand-to-hand contact because it is sticky and thus more likely to be transferred onto surfaces (and dust) or people. Peanut protein persists on table surfaces and sofa-pillow dust, despite usual cleaning measures,^26^ and thus might be an important source of exposure.

Although our study focused on peanut sensitization and allergy, *FLG* loss-of-function mutations might confer susceptibility to environmental exposure to other food allergens in dust, such as fish, egg, and cow's milk.^45^ The dual-allergen-exposure hypothesis postulates that food allergy develops through transcutaneous exposure to allergen through a disrupted skin barrier, whereas oral exposure leads to tolerance induction.^38^ Our findings of a dose-response effect for peanut allergen in dust on the development of peanut allergy in children genetically predisposed to a skin barrier defect support this hypothesis. Furthermore, our study raises the intriguing possibility of identifying a group of children with *FLG* loss-of-function mutations and targeting them in interventional studies through early environmental modification.

**Clinical implications**

Children with *FLG* loss-of-function mutations are at an increased risk of peanut sensitization and allergy if they are exposed to peanut antigen in household dust in early life. Interventional studies to assess a causal relationship are required.

## Figures and Tables

**Table E1:** GEE for peanut sensitization using the quasilikelihood under independent model criterion goodness-of-fit analyses

	GEE for peanut SPT sensitization adjusted for clustering at age 8 and 11 y (n = 584)					GEE for peanut CRD sensitization adjusted for clustering at age 8 and 11 y (n = 437)					LR for peanut allergy at age 8 y, 11 y, or both (n = 577)				
	No.∗	OR	95% CI	*P* value	QIC§	No.†	OR	95% CI	*P* value	QIC§	No.‡	OR	95% CI	*P* value	AIC§
Combined *FLG* loss-of-function mutation	584	3.5	1.5-8.3	**<.01**	386.6	437	4.0	1.4-11.4	**<.01**	215.3	577	2.5	0.8-7.9	.11	175.6
Age at assessment (8 or 11 y)		0.8	0.5-1.1	.10			0.9	0.6-1.4	.69			NA			

Combined *FLG* loss-of-function mutation	584	3.6	1.5-8.2	**<.01**	386.5	437	3.9	1.4-10.9	**<.01**	215.9	577	2.6	0.8-7.9	.11	176.8
Peanut protein in dust (*ln* transformed μg/g)‖		1.2	0.9-1.7	.15			1.3	0.90-1.8	.19			1.2	0.8-1.8	.35	
Age at assessment (8 or 11 y)		0.7	0.5-1.1	.10			0.9	0.6-1.4	.65			NA			

Combined *FLG* loss-of-function mutation	584	2.8	0.8-9.8	.17	373.2	437	2.6	0.6-11.1	.20	208.2	577	2.3	0.6-8.4	.21	175.5
Peanut protein in dust (*ln* transformed μg/g)‖		0.9	0.6-1.3	.58			0.9	0.5-1.4	.51			1.0	0.6-1.6	.97	
Interaction FLG*peanut in dust		4.3	1.4-12.8	**.01**			4.0	1.4-11.4	**.01**			2.5	0.9-7.1	.08	
Age at assessment (8 or 11 y)		0.7	0.5-1.1	.10			0.9	0.6-1.4	.66			NA			

Combined *FLG* loss-of-function mutation	516	2.1	0.5-8.4	.30	307.1	396	1.2	0.2-7.2	.78	177.0	511	1.1	0.2-5.1	.91	132.4
Peanut protein in dust (*ln* transformed μg/g)‖		0.9	0.6-1.3	.59			0.9	0.6-1.5	.75			1.1	0.6-1.9	.83	
Interaction FLG*peanut in dust		5.5	2.0-14.7	**.001**			6.0	2.2-16.2	**<.001**			3.7	1.3-10.7	**.02**	
Egg SPT sensitization at age 3 y		15.9	4.4-57.8	**<.001**			26.3	5.3-130.2	**<.001**			35.9	10.1-127.7	**<.001**	
Age at assessment (8 or 11 y)		0.7	0.5-1.1	.13			0.9	0.6-1.6	.81			NA			

Combined *FLG* loss-of-function mutation	516	1.3	0.3-5.8	.71	279.4	396	0.9	0.2-5.3	.93	167.9	511	0.8	0.2-3.9	.81	129.3
Peanut protein in dust (*ln* transformed μg/g)‖		0.9	0.6-1.3	.52			0.9	0.6-1.4	.67			1.0	0.6-1.8	.89	
Interaction FLG*peanut in dust		4.3	1.7-11.0	**<.01**			4.8	1.8-12.6	**<.01**			3.1	1.1-8.9	**.04**	
Egg SPT sensitization at age 3 y		8.6	2.2-33.6	**<.01**			13.5	2.3-79.2	**<.01**			20.4	5.5-76.3	**<.001**	
Atopic eczema during infancy		7.6	2.5-23.3	**<.001**			5.4	1.2-24.2	**<.01**			4.1	1.2-14.1	**.03**	
Age at assessment (8 or 11 y)		0.7	0.4-1.1	.12			1.0	0.6-1.7	.91			NA			

Values in boldface are significant.

*AIC*, Akaike information criterion; *LR*, penalized logistic regression methodology; *NA*, not applicable; *QIC*, quasilikelihood under independent model criterion.

∗†‡ White children enrolled in the MAAS with available sofa dust within the first year of life, successful *FLG* genotyping, and peanut SPT* or CRD† sensitization or peanut allergy‡ assessment.

‖ Peanut protein levels in dust less than the LLQ were assigned an LLQ/2 calculation.

§ Reductions in QIC (GEE) and AIC (LR) values denote improved goodness-of-fit of statistical model.
