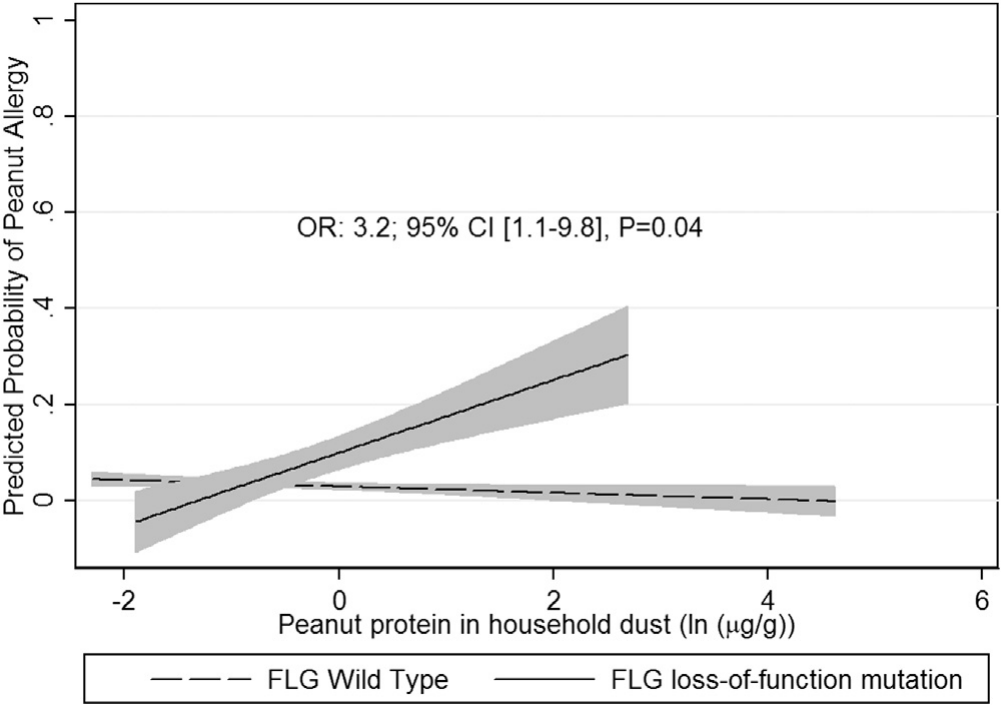# Correction

**DOI:** 10.1016/j.jaci.2014.10.024

**Published:** 2014-12

With regard to the article in the October 2014 issue entitled “Peanut allergy: Effect of environmental peanut exposure in children with filaggrin loss-of-function mutations” (J Allergy Clin Immunol 2014;134:867-75.e1), the authors report that the key showing the definitions in Fig 3 was inadvertently omitted. The figure appears below with the corrected key: FLG Wild Type (dashed line) and FLG loss-of-function mutation (solid line). The authors regret the error.

**Figure:**